# Complete genome sequence of *Pseudomonas fluorescens* strain PICF7, an indigenous root endophyte from olive (*Olea europaea* L.) and effective biocontrol agent against *Verticillium dahliae*

**DOI:** 10.1186/1944-3277-10-10

**Published:** 2015-02-06

**Authors:** Pedro Manuel Martínez-García, David Ruano-Rosa, Elisabetta Schilirò, Pilar Prieto, Cayo Ramos, Pablo Rodríguez-Palenzuela, Jesús Mercado-Blanco

**Affiliations:** 1 Instituto de Hortofruticultura Subtropical y Mediterránea “La Mayora”, Universidad de Málaga - Agencia Estatal Consejo Superior de Investigaciones Científicas (IHSM-UMA-CSIC), Área de Genética, Facultad de Ciencias, Málaga, Spain; 2 Departmentos de Potección de Cultivos y, Campus ‘Alameda del Obispo’ s/n, Apartado 4084, 14080 Córdoba, Spain; 3 Departmentos de Mejora Genética Vegetal, Instituto de Agricultura Sostenible (CSIC), Campus ‘Alameda del Obispo’ s/n, Apartado 4084, 14080 Córdoba, Spain; 4 Centro de Biotecnología y Genómica de Plantas (UPM- INIA), Campus de Montegancedo 28223, Pozuelo de Alarcón, Madrid, Spain

**Keywords:** *Pseudomonas fluorescens*, Olive, Endophyte, Biocontrol, Verticillium wilt, Siderophores, Detoxification systems

## Abstract

*Pseudomonas fluorescens* strain PICF7 is a native endophyte of olive roots. Previous studies have shown this motile, Gram-negative, non-sporulating bacterium is an effective biocontrol agent against the soil-borne fungus *Verticillium dahliae*, the causal agent of one of the most devastating diseases for olive (*Olea europaea* L.) cultivation. Here, we announce and describe the complete genome sequence of *Pseudomonas fluorescens* strain PICF7 consisting of a circular chromosome of 6,136,735 bp that encodes 5,567 protein-coding genes and 88 RNA-only encoding genes. Genome analysis revealed genes predicting factors such as secretion systems, siderophores, detoxifying compounds or volatile components. Further analysis of the genome sequence of PICF7 will help in gaining insights into biocontrol and endophytism.

## Introduction

*Pseudomonas fluorescens* PICF7 is a native colonizer of olive (*Olea europaea* L.) roots and an *in vitro* antagonist of the soil-borne fungal phytopathogen *Verticillium dahliae* Kleb. [1], the causal agent of Verticillium wilts in a large number of plant species [2]. This strain has been demonstrated to be an effective BCA against Verticillium wilt of olive [1,3], one of the most important biotic constraints for olive cultivation [4]. Moreover, strain PICF7 is able to display an endophytic lifestyle within olive root tissues under different experimental conditions [3,5,6] and induces a broad range of defence responses at both local (roots) and systemic (above-ground organs) level, as well as to activate diverse transcription factors known to be involved in systemic defence responses [7,8]. Accordingly, a recent study has shown the ability of PICF7 to influence the establishment of the pathogen *Pseudomonas savastanoi* pv. savastanoi in olive stems and to affect the normal development of olive knots [9], its associated disease [10].

In this report, we summarize the complete genome sequence and annotation of PICF7. We also describe its genomic properties, highlighting genes encoding plant-associated factors, colonization abilities and well-known bacterial biocontrol traits. The genome sequencing of PICF7 and its comparison with related published genomes will provide a framework for further functional studies of its rhizosphere competence, biocontrol effectiveness and endophytic lifestyle.

### Classification and features

**Figure 1:**
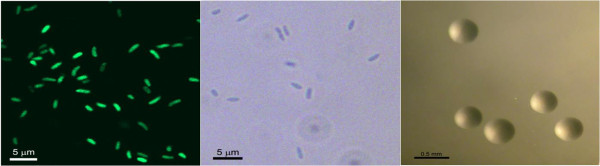
**Image of *P. fluorescens *PICF7 cells using confocal laser scanning (Left) and phase-contrast (Centre) microscopy (CLSM and PCM, respectively) and the appearance of colony morphology after 24 h growing on KB agar medium at 28°C (Right).** CLSM image was obtained using a PICF7 derivative carrying a plasmid with an enhanced green fluorescent protein (EGFP) [5].

*P. fluorescens* PICF7 is a motile, Gram-negative, non-sporulating rod in the order *Pseudomonadales* of the class *Gammaproteobacteria*. Rod-shaped cells are approximately 0.5 μm in width and 2.0-2.5 μm in length (Figure 1 Left and Centre). The strain is moderately fast-growing, forming 2 mm colonies within 2-3 days at 28°C. Colonies formed on King’s B (KB) [11] agar plates are yellow-green opaque, domed and moderately mucoid with smooth margins (Figure 1 Right).

PICF7 was isolated from the roots of healthy nursery-produced olive plants cv. Picual in Córdoba province (Southern Spain) [1]. It grows in complex media such as LB [12] or KB, as well as in minimal media such as Standard Succinate Medium (SSM; pH 7.0) [13]. Even though the optimal growth temperature is 28°C, PICF7 can also slightly replicate at 5°C in liquid LB and KB. However, growth at 37°C was not observed in these culturing media after 24 h. The bacterium is an efficient colonizer of the olive rhizosphere [1] and displays an endophytic lifestyle [3,5,6]. It does not cause any deleterious effect on its original host (olive) [1,5,9]. Strain PICF7 has natural resistance to kanamycin (50 mg/L) and nalidixic acid (25 mg/L), and it is possible to develop spontaneous rifampicin-resistant mutants [1].

**Figure 2:**
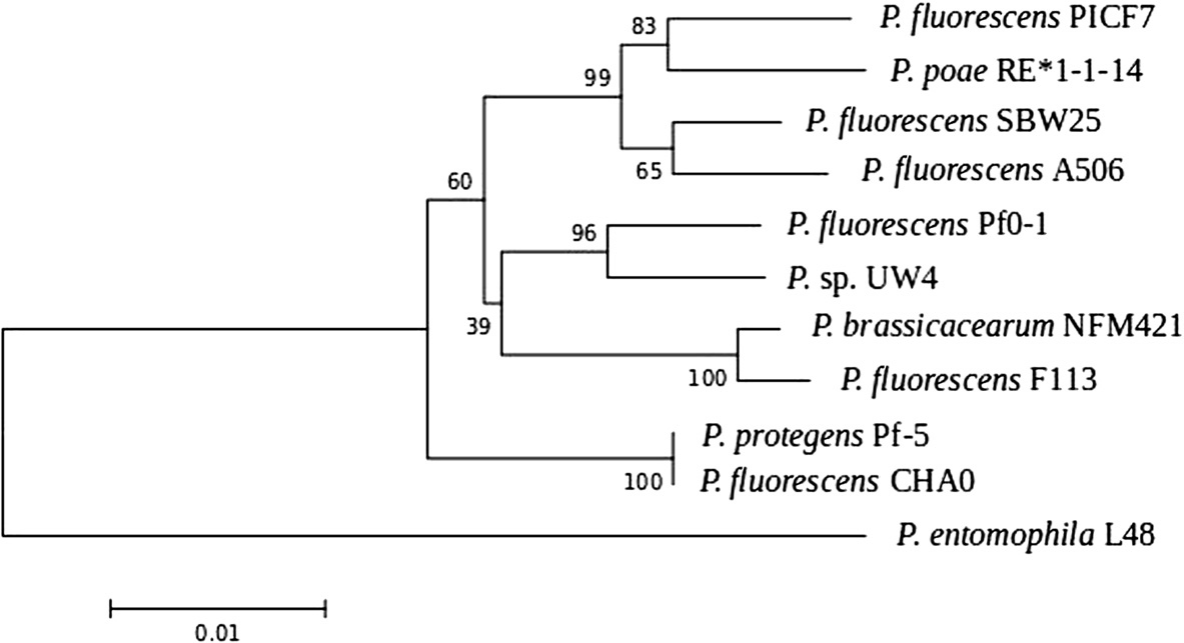
**Phylogenetic tree highlighting the position of *P. fluorescens *strain PICF7 relative to its closest *Pseudomonas *strains for which complete genomes are available.***P. entomophila* strain L48 was used as an outgroup. For the construction of the tree, five protein-coding house-keeping genes were first aligned, namely: argF, atpA, nusA, pyrH and rpoH. Then, Maximum Likelihood method based on the JTT (Jones-Taylor-Thornton) matrix-based model [29] was used. The percentage of trees in which the associated taxa clustered in the bootstrap test (1000 replicates) is shown next to the branches [30].

Minimun Information about the Genome Sequence (MIGS) of *P. fluorescens* PICF7 is summarized in Table 1, and its phylogenetic position is shown in Figure 2.

**Table 1:** **Classification and the general features of *Pseudomonas fluorescens *** **PICF7 according to the MIGS recommendations [**[14]**]**

**MIGS ID**	**Property**	**Term**	**Evidence code**^**a**^
		Domain *Bacteria*	TAS [15]
		Phylum *Proteobacteria*	TAS [16]
		Class *Gammaproteobacteria*	TAS [17,18]
	Classification	Order *Pseudomonadales*	TAS [19,20]
		Family *Pseudomonadaceae*	TAS [21,22]
		Genus *Pseudomonas*	TAS [21-25]
		Species *Pseudomonas fluorescens*	TAS [26,27]
		Strain PICF7	TAS [1,5]
	Gram stain	Negative	TAS [26]
	Cell shape	Rod-shaped	TAS [26]
	Motility	Motile	TAS [26]
	Sporulation	None	NAS
	Temperature range	Mesophilic	IDA
	Optimum temperature	28°C	IDA
MIGS-22	Oxygen	Aerobic	IDA, TAS [26]
	Carbon source	Heterotrophic	IDA, TAS [26]
	Energy metabolism	Chemoorganotrophic	NAS
MIGS-6	Habitat	Soil, olive root-associated	TAS [1,5]
MIGS-6.3	Salinity	NaCl 1-4%	IDA
MIGS-10	Extrachromosomal elements	None	IDA
MIGS-11	Estimated size	6.14 Mb	IDA
			
MIGS-15	Biotic relationship	Rhizospheric, root endophytic	TAS [1,3,5,6]
MIGS-14	Pathogenicity	Non-pathogenic	TAS [1,3,5]
	Host	*Olea europaea*	TAS [1]
	Host taxa ID	4146	
	Isolation source	Root	TAS [1]
	Biosafety level	1	NAS
MIGS-4	Geographic location	Córdoba, Spain	TAS [1]
MIGS-5	Sample collection time	1998	TAS [1]
MIGS-4.1	Latitude	37°41′28″N	NAS
MIGS-4.2	Longitude	4°28′58″W	NAS
MIGS-4.4	Altitude	230 m.a.s.l	NAS

^a^Evidence codes - IDA: Inferred from Direct Assay (first time in publication); TAS: Traceable Author Statement (a direct report exists in the literature); NAS: Non-traceable Author Statement (not directly observed for the living, isolated sample, but based on a generally accepted property of the species, or anecdotal evidence). These evidence codes are from the Gene Ontology project [28].

## Genome sequencing and annotation

### Genome project history

*P. fluorescens* strain PICF7 was selected for sequencing due to its ability to exert biocontrol against Verticillium wilt of olive [1,3] and to develop an endophytic lifestyle within olive root tissues [5,6]. The genome project is deposited in the Genomes OnLine Database [31] and the NCBI BioProject database. The finished genome sequence is in GenBank. A summary of the project information is shown in Table 2.

**Table 2:** Project information

**MIGS ID**	**Property**	**Term**
MIGS-31	Finishing quality	Finished
MIGS-28	Libraries used	Three libraries of 500 bp, 2,000 bp and 6,000 bp, respectively
MIGS-29	Sequencing platforms	Solexa
MIGS-31.2	Fold coverage	200 x
MIGS-30	Assemblers	SOAPdenovo 1.05
MIGS-32	Gene calling method	NCBI Prokaryotic Genome Annotation Pipeline
	Locus Tag	PFLUOLIPICF7
	Genbank ID	CP005975
	GenBank Date of Release	May 31, 2017
	GOLD ID	Gi0079402
	BIOPROJECT	PRJNA203247
	NCBI taxon ID	1334632
	Project relevance	Plant-bacteria interaction, Model for endophytic lifestyle, Agricultural, Environmental

### Growth conditions and DNA isolation

*P. fluorescens* strain PICF7 was grown in 50 ml of LB medium and incubated for 16 h at 28°C. After this period of time, the OD600 of the culture was 1.2. Serial dilutions from this culture and plating on LB plates yielded 2.8 × 10^8^ CFU/mL of a pure bacterial culture (colonies showed uniform morphology and kanamycin resistance). The culture was divided into two 25-ml aliquots and total genomic DNA was extracted using the ‘Jet-Flex genomic DNA purification’ kit (Genomed GmbH, Löhne, Germany), according to the manufacturer’s indications. DNA samples were further purified by extraction with phenol:chloroform and precipitation with ethanol. DNA quality and quantity were checked by agarose gel electrophoresis, spectrophotometry using a ND-1000 spectrophotometer (NanoDrop Technologies, Wilmington, DE), and digestion with different restriction enzymes. Two DNA aliquots (0.6 μg/μL, ~20 μg each) were sent in a dry ice container to the sequencing service.

### Genome sequencing and assembly

The genome of PICF7 was sequenced at the Beijing Genomics Institute (BGI) using Solexa paired-end sequencing. Draft assemblies were based on 3,482,351 reads with a length of 500 bp resulting in 1,200 Mb, 2,456,221 reads with a length of 2,000 bp resulting in 1,209 Mb and 1,924,515 reads with a length of 6,000 bp resulting in 1,309 Mb. The SOAPdenovo 1.05 software package [32-34] developed by BGI was used for sequence assembly and quality assessment.

### Genome annotation

Automatic annotation was performed using the NCBI Prokaryotic Genome Annotation Pipeline. Identification of known type III effectors effectors was conducted by BLASTP searches of the effectors described in http://pseudomonas-syringae.org/ against the proteome of PICF7. Functional annotation was performed by aligning the predicted protein sequences against the COG PSSM of the CDD using RPS-BLAST. Hits with an E-value < = 0.001 were first retained. Then, only the best hit was selected for each protein. Signal peptides and transmembrane helices were predicted using SignalP [35,36] and TMHMM [37,38], respectively.

### Genome properties

**Figure 3:**
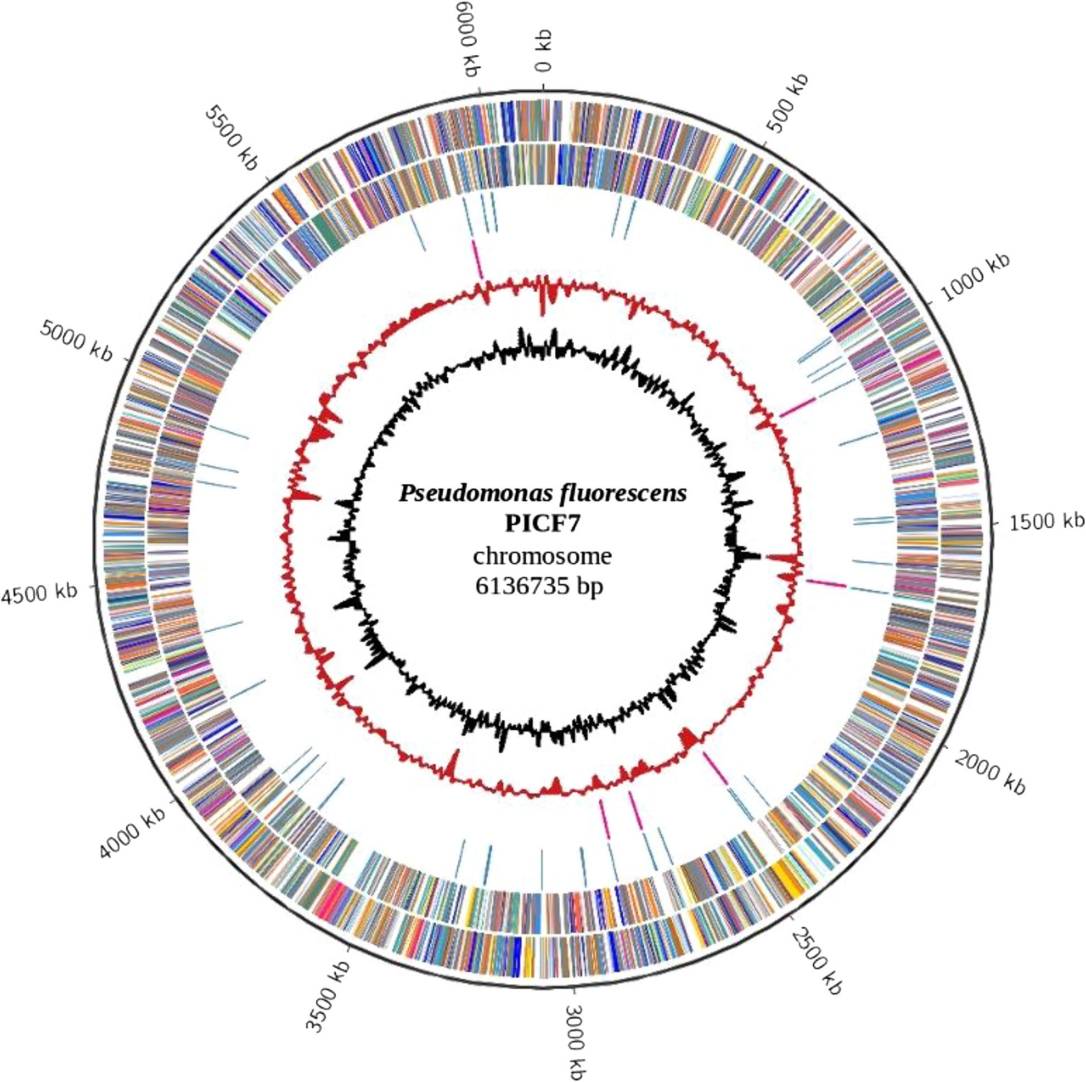
**Graphical map of the chromosome.** From outside to the centre: genes on forward strand (coloured by COG categories), genes on reverse strand (coloured by COG categories), RNA genes: tRNAs - blue, rRNAs – pink, G + C in relation to the mean G + C in 2 kb windows and trinucleotide distribution in 2 kb windows. The latter was defined as the χ2 statistic on the difference between the trinucleotide composition of 2 kb windows and that of the whole chromosome.

The genome of PICF7 is composed of one circular chromosome of 6,136,735 bp with an average GC content of 60.4% (Table 3 and Figure 3), which is similar to that of other *P. fluorescens* strains. Among the 5,655 predicted genes, 5,567 were identified as protein coding genes. Of the last, 4,573 (82.1%) were assigned a putative function, while the other 994 (17.9%) were designated as hypothetical proteins. The classification of CDSs into functional categories according to the COG (Clusters of Orthologous Groups) [39,40] database is summarized in Table 4.

**Table 3:** Genome statistics

**Attribute**	**Genome (total)**	
	**Value**	**% of total**
Genome size (bp)	6,136,735	100
DNA coding region (bp)	5,439,499	88.6
DNA G+C content (bp)	3,706,588	60.4
DNA scaffolds	1	-
Total genes	5,655	100
Protein-coding genes	5,567	98.4
RNA genes	88	1.6
Pseudo genes	30	0.8
Genes in internal clusters	NA	-
Protein-coding genes with function prediction	4,573	82.1
Protein-coding genes assigned to COGs	4,581	82.3
Proteins with signal peptides	644	11.6
Proteins with transmembrane helices	1,319	23.7
CRISPR repeats	NA	-

**Table 4:** Number of genes associated with general COG functional categories

**Code**	**Value**	**% of total**^**a**^	**Description**
J	200	3.59	Translation
A	1	0.02	RNA processing and modification
K	501	9	Transcription
L	156	2.8	Replication, recombination and repair
B	5	0.09	Chromatin structure and dynamics
D	41	0.74	Cell cycle control, mitosis and meiosis
Y	-	-	Nuclear structure
V	67	1.2	Defense mechanisms
T	366	6.57	Signal transduction mechanisms
M	267	4.8	Cell wall/membrane biogenesis
N	162	2.9	Cell motility
Z	-	-	Cytoskeleton
W	-	-	Extracellular structures
U	153	2.75	Intracellular trafficking and secretion
O	177	3.18	Posttranslational modification, protein turnover, chaperones
C	280	5.03	Energy production and conversion
G	307	5.51	Carbohydrate transport and metabolism
E	554	9.95	Amino acid transport and metabolism
F	96	1.72	Nucleotide transport and metabolism
H	196	3.52	Coenzyme transport and metabolism
I	219	3.93	Lipid transport and metabolism
P	301	5.41	Inorganic ion transport and metabolism
Q	151	2.71	Secondary metabolites biosynthesis, transport and catabolism
R	592	10.63	General function prediction only
S	446	8.01	Function unknown
-	986	17.7	Not in COGs

^a^The total is based on the total number of protein coding genes in the annotated genome.

### Insights from the genome sequence

The genome contains a complete canonical type III secretion system and two known effector proteins, namely, AvrE1 and HopB1. In addition, two complete type VI secretion system (T6SS) clusters were identified. T6SS has been described to promote antibacterial activity against a wide range of competitor bacteria [41]. PICF7 genome also encodes gene clusters for the synthesis of the siderophores pyochelin and pyoverdine and the hemophore HasAp. A repertoire of cell adhesion proteins has been also identified, including two filamentous hemagglutinin proteins and several fimbrial proteins clustered together with a number of pilus assembly proteins. Notably, two genes have been found to show high similarity with *attC* and *attG* genes from *Agrobacterium*, whose mutation leads to lack of attachment on tomato, carrot, and *Bryophyllum daigremontiana*[42].

It is worth mentioning the presence of genome components presumably involved in the synthesis of detoxifying compounds. Such is the case of two clusters containing genes for copper resistance and for production of a cbb(3)-type cytochrome C oxidase, respectively. An ortholog of the gene that codes for Dps, a ferritin-like protein reported to protect plant-associated bacteria against oxidative stress [43], has also been found. Additional identified traits involved in detoxification are orthologs of catalase KatB and hydroperoxidase KatG, which detoxify plant-produced H_2_O_2_[44], and a gene coding for a proline iminopeptidase, which has been shown to have dealanylating activity toward the antibiotic ascamycin [45]. A gene predicting a salycilic hydroxylase has been also identified in PICF7 genome. This gene could be involved in the degradation of the plant defence hormone salicylic acid, thus disrupting the systemic response against colonizing bacteria. In addition, all genes required for biosynthesis of the exopolysaccharide alginate [46] are present in a gene cluster.

Genes predicting volatile components are present in PICF7 genome as well. Volatile components have been shown to act as antibiotics and to induce plant growth [47,48]. An example is hydrogen cyanide (HCN), an inorganic compound with antagonistic effects against soil microbes [49]. Orthologs of genes required for the biosynthesis of other volatile components such as 2,3-butanediol and acetoin were also found. Further genome analysis revealed other factors presumably involved in the endophytic fitness of PICF7. Such is the case of enzymes like a cellulase and a phytase, as well as the gene coding for aminocyclopropane-1-carboxylate deaminase suggested to be key in the modulation of ethylene levels in plants by bacteria [50].

## Conclusions

In this report we describe the complete genome sequence of Pseudomonas fluorescens strain PICF7, a “Pseudomonadales” in the order Gammaproteobacteria that was originally isolated from the roots of healthy nursery-produced olive plants cv. Picual in Córdoba province, Spain. This strain was selected for sequencing based on its ability to exert biocontrol against Verticillium wilt of olive and to develop an endophytic lifestyle within olive root tissues. Such properties likely have origins in a repertoire of genes including a putative T3SS, two putative T6SS, and several genes presumably implicated in siderophore production. It also has a collection of genes predicting adhesion proteins, detoxifying compounds, volatile components and enzymes such as a cellulase, aphytase and a deaminase. Further functional studies and comparative genomics with related isolates will provide insights into biocontrol and endophytism.

## Competing interests

The authors declare that they have no competing interests.

## Authors’ contributions

JMB conceived and designed the experiments. DRR, ES and PP performed the experiments. PMMG performed the annotation and sequence homology searches. PMMG, CR, JMB and PRP wrote the manuscript. All authors commented on the manuscript before submission. All authors read and approved the final manuscript.
